# Viral non-coding RNA inhibits HNF4α expression in HCV associated hepatocellular carcinoma

**DOI:** 10.1186/s13027-015-0014-0

**Published:** 2015-07-08

**Authors:** Zhao Wang, Kristin Ceniccola, Liliana Florea, Bi-Dar Wang, Norman H. Lee, Ajit Kumar

**Affiliations:** Department of Biochemistry, The George Washington University, Washington, DC USA; Department of Pharmacology, and Program in Molecular Oncology, The George Washington University, Washington, DC USA; McKusick-Nathans Institute of Genetic Medicine, The Johns Hopkins University, Baltimore, MD USA

## Abstract

**Background:**

Hepatitis C virus (HCV) infection is an established cause of chronic hepatitis, cirrhosis and hepatocellular carcinoma (HCC); however, it is unclear if the virus plays a direct role in the development of HCC. Hepatocyte nuclear factor 4α (HNF4α) is critical determinant of epithelial architecture and hepatic development; depletion of HNF4α is correlated with oncogenic transformation. We explored the viral role in the inhibition of HNF4α expression, and consequent induction of tumor-promoting genes in HCV infection-associated HCC.

**Methods:**

Western blot analysis was used to monitor the changes in expression levels of oncogenic proteins in liver tissues from HCV-infected humanized mice. The mechanism of HNF4α depletion was studied in HCV-infected human hepatocyte cultures *in vitro*. Targeting of HNF4α expression by viral non-coding RNA was examined by inhibition of Luciferase HNF4α 3’-UTR reporter. Modulation of invasive properties of HCV-infected cells was examined by Matrigel cell migration assay.

**Results:**

Results show inhibition of HNF4α expression by targeting of HNF4α 3’-UTR by HCV-derived small non-coding RNA, vmr11. Vmr11 enhances the invasive properties of HCV-infected cells. Loss of HNF4α in HCV-infected liver tumors of humanized mice correlates with the induction of epithelial to mesenchymal transition (EMT) genes.

**Conclusions:**

We show depletion of HNF4α in liver tumors of HCV-infected humanized mice by HCV derived small non-coding RNA (vmr11) and resultant induction of EMT genes, which are critical determinants of tumor progression. These results suggest a direct viral role in the development of hepatocellular carcinoma.

## Background

Hepatitis C virus (HCV) infection is a major cause of chronic hepatitis, liver cirrhosis, and hepatocellular carcinoma (HCC) [1]. Each year over a half million new cases of HCC are diagnosed worldwide, with approximately 20,000 new cases in the United States alone; HCC related to HCV infection is the most rapidly rising cause of cancer-related deaths in the United States [2]. However, patients who are treated and achieve a sustained virologic response have a markedly reduced risk of developing HCC. We examined the possible role of viral factors in the development of HCC using MUP-uPA/SCID/Bg mice engrafted with human hepatocytes and infected with HCV [3]. We describe results showing viral non-coding RNA vmr11 directed loss of HNF4α and subsequent induction of EMT genes in HCV-infected HCC in the humanized mouse model. We used human primary hepatocyte cultures [4] to study the mechanism of HNF4α depletion by the HCV-derived small non-coding RNA.

Hepatocyte nuclear factor 4α (HNF4α) is member of the steroid hormone receptor superfamily of nuclear transcription factors with critical roles in hepatocyte differentiation, liver development and the maintenance of epithelial architecture [5, 6]. Liver-specific inactivation of HNF4α is linked to hepatomegaly, abnormal deposition of glycogen, cholesterol and lipid metabolism [7–9]. HNF4α contributes to the regulation of a large fraction of liver transcriptome by binding to promoters of actively transcribed genes [10]. HNF4α plays a critical role in the maintenance of hepatic epithelium by suppressing expression of epithelial to mesenchymal transition (EMT) genes [11]. Loss of HNF4α has been shown to result in the induction of EMT genes and oncogenic transformation [11–13]. We sought to understand the mechanism of HNF4α depletion and regulation of tumor development by investigating whether HCV triggers the loss of HNF4α and consequent induction of tumorigenic genes in HCC.

A normal level of HNF4α protein is critical in maintaining hepatic epithelial architecture and liver function. Depletion of HNF4α is a critical determinant of epithelial-to-mesenchymal transition, characterized by loss of the epithelial marker E-Cadherin and induction of the mesenchymal marker Vimentin during tumor progression. Here we describe results showing that loss of HNF4α is triggered by HCV-derived 22 nucleotide vmr11 RNA [14]. Vmr11 RNA is sufficient to confer HCV-infected hepatocytes with invasive properties. Sustained loss of HNF4α in HCV infection-associated liver tumor down-regulated *E-Cadherin* and induced the expression of Vimentin and EMT regulatory genes *Snail*, *TGF-*β and HMGA2. These results support a direct viral role of HCV in the development of hepatocellular carcinoma by the targeting of HNF4α expression with viral non-coding RNA, vmr11.

## Methods

### Transfection of PPH cultures with HCV1a genomic RNA

HCV genomic RNA was prepared by linearizing pCV-H77c (HCV genotype 1a cDNA) plasmid with Xba1, followed by run-off transcription with T7 Ribomax Express (Promega). 5 X 10^5^ human primary hepatocytes (PPH) were transfected with 1 μg of the H77 RNA using Fugene 6 (Promega) procedure [14]. Vmr11 oligonucleotides transfections were carried out using Lipofectamine® 2000 Transfection Reagent (Invitrogen™), with 50nM vmiR-11 mimic or, 50nM vmiR-11 (LNA-modified) antagomir. The vmiR-11 oligonucleotides were transfected twice at 24-h intervals and the cells were harvest at 48 h post-transfection.

### RNA extraction

Total RNA from human hepatocyte cultures or the liver tissues were lysed by TRIzol® Reagent (Ambion) according to the manufacturer’s protocol. Briefly, tissue samples were cut into small piece and rinsed in 1 mL of TRIzol® Reagent. Liver tissues were homogenized and total RNA was separated with 0.2 mL chloroform and precipitated with 0.5 mL Iso-propanol. The RNA pellet was washed with 75 % ethanol. RNA concentration was measured using the NanoDrop 1000 Spectrophotometer.

### Reverse transcription and quantitative real time PCR

First strand cDNA was synthesized by QuantiTect Reverse Transcription protocol (QIAGEN) with 1 microgram RNA samples. The cDNA products were diluted 50 fold for qRT-PCR assay. RT-PCR was performed using iTaq Universal SYBR Green Supermix with Rox (Bio-Rad) on ABI 7300 Real Time PCR system. Primers used were as follows: *HNF4α-forward*: 5'-TGTCC CGACA GATCA CCTC- 3'; *HNF4α-reverse*: 5'- CACTC AACGA GAACC AGCAG- 3'; *18 s rRNA-forward*: 5'- GTAAC CCGTT GAACC CCATT -3', *18 s rRNA-reverse*: 5' -CCATC CAATC GGTAG TAGCG -3'. Virus replication was monitored either by estimating genomic equivalents (GE) of HCV RNA (by nested PCR [4]) from virus particles recovered from the culture medium, or by immune blotting for viral antigen, NS5A or HCV core [4].

### Protein extraction and immunoblotting

PPH cultures (treated as indicated) were lysed with RIPA buffer supplemented with 1 X protease inhibitor cocktail (Roche). 10 μg of protein was analyzed on 10 % precast Mini-PROTEIN gel (BIO-RAD). Proteins were transferred to PVDF membrane, blocked with 5 % non-fat dry milk and probed with antibodies as indicated. HRP conjugated anti-rabbit, anti-mouse, anti-goat antibodies (from Abcam; SuperSignal West Dura Chemiluminescent Substrate) were visualized under BIO-RAD ChemiDoc™ XRS+ System. Images were analyzed (by Image Lab™ Software developed by BIO-RAD), and the protein amounts estimated by normalizing to β-Actin as internal control [14].

Liver tissues used for Western blot analysis were from human hepatocyte engrafted MUP-uPA/SCID/Bg mice, either uninfected (controls) or HCV infection-induced liver tumors. The chimeric mouse liver tissues for these studies were provided by Drs. Tesfaye and Feinstone [3]. The liver tissues were lysed with RIPA buffer supplemented with protease inhibitor cocktail (Roche). 10 μg of protein was analyzed on 10 % precast Mini-PROTEIN gel (BIO-RAD). The gels were transferred to PVDF membrane, blocked with 5 % non-fat dry milk and probed with antibodies (Abcam) against the proteins as indicated. HRP conjugated anti-rabbit, or anti-goat antibodies (Abcam; SuperSignal West Dura Chemiluminescent Substrate) were visualized using BIO-RAD ChemiDoc™ XRS+ System. Western blots were analyzed by Image Lab™ Softwar developed by BIO-RAD; and the protein quantitation was based on β-Actin internal (loading) control.

### Matrigel assay

Primary human hepatocytes (1 x 10^4^) were seeded in the top well of a Matrigel-coated invasion chamber (BD Biosciences) in DMEM containing 0.1 % serum. The bottom well was filled with 750 μL DMEM containing 10 % serum as a chemoattractant. After 48 h, non-invading cells were scraped from the upper side of the insert using a cotton swab. Invading cells on the bottom of the insert were fixed and stained with Diff-Quick Stain (IMEB, Inc., San Marcos, CA) according to manufacturer’s instructions. The total number of invading cells was counted for each insert under a light microscope.

## Results

### Loss of HNF4α in HCV-infection associated hepatocellular carcinoma

We attempted to establish a link between HCV infection and loss of HNF4α in HCV infection associated HCC using a recently reported [3] chimeric mouse model. Earlier studies with transgenic mice explored a direct role of HCV proteins in promoting hepatocarcinogenesis [15, 16]. Transgenic mice however, do not model HCV infection associated liver cancer, nor are the tumors of human origin.

Mercer et al [17] reported successful HCV infection in chimeric mice that had much of their livers replaced by human liver. These immunodeficient (SCID/bg) mice carry tandem copies of urokinase plasminogen activator (uPA) gene under the control of albumin promoter (Alb-uPA). In an improved version of uPA transgenic mouse, the tandem array of uPA genes was put under the control of major urinary protein (MUP) promoter [18]; MUP is not expressed until after three weeks of life. The MUP-uPA mice were crossed with SCID/bg mice and triply homozygous, MUP-uPA/SCID/bg mice were recovered; these mice are robust, breed well with high infant survival, and can be transplanted with human hepatocytes and infected with (most genotypes of) HCV [3]. The control animals in this study were engrafted but not infected with HCV, and remained healthy for up to eleven months.

**Fig. 1:**
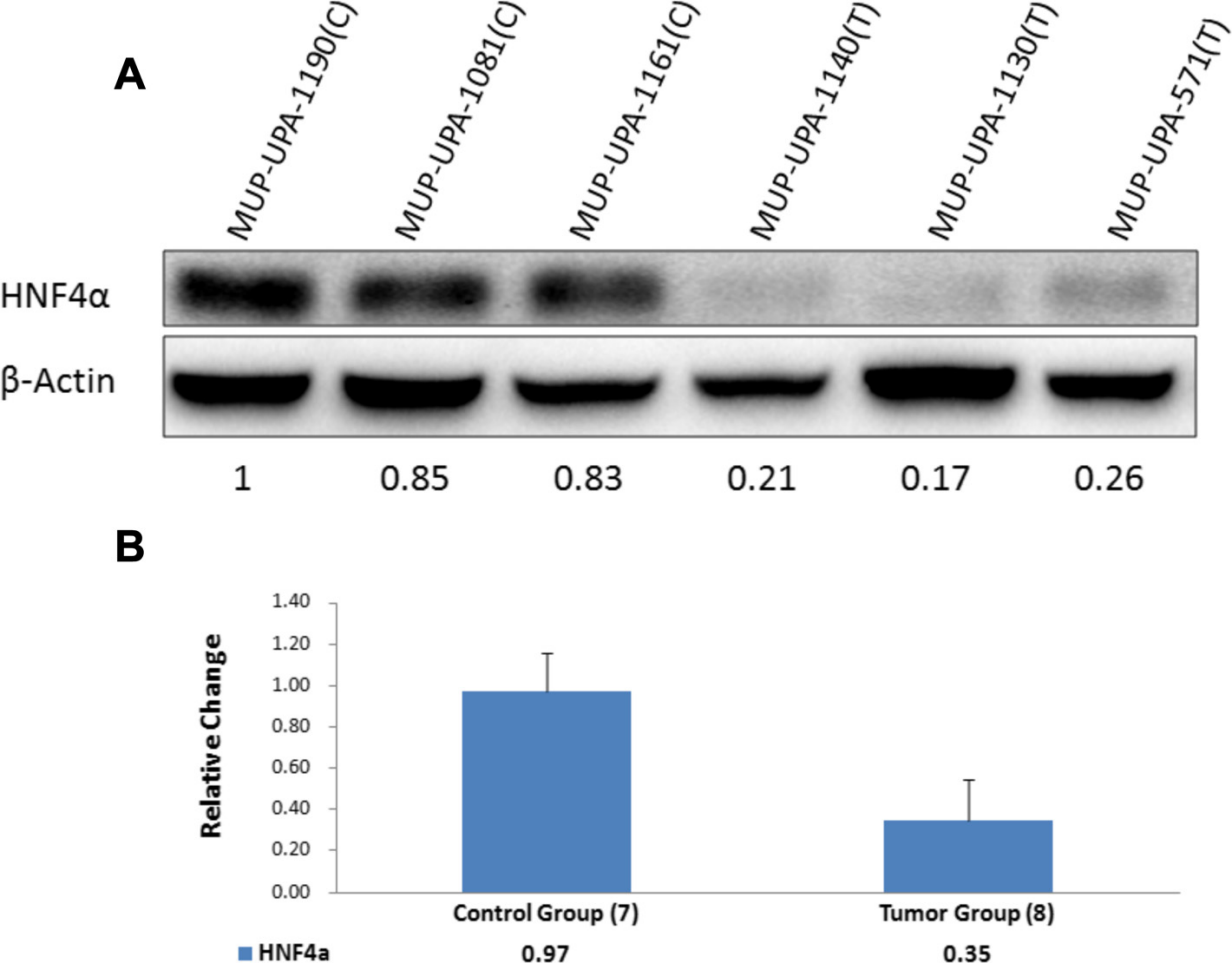
*HNF4*α *protein in HCV-infection associated liver tumor:* (**a**). Representative Western blots of three controls (**c**), and three liver tumors (T) are shown. Change in HNF4α α protein level was normalized to β-Actin loading control; relative values are indicated underneath each lane. **b** Quantitative change in HNF4α protein levels was estimated by similar Western blot analyses of 7 controls and 8 liver tumors from HCV infected chimeric mice. The relative values of HNF4α shown were based on Western blots run in triplicates (mean ^+/-^ SE) (*p < 0.01)

Representative Western blots of HNF4α protein in liver tissues of three tumor- bearing and three control mice are shown in Fig. 1a. Quantitative assessment of HNF4α protein in HCV-infected liver tumors was made from similar Western blot analyses of seven controls and eight tumor tissues from human hepatocyte-engrafted and HCV-infected MUP-uPA/SCID/Bg mice (Fig. 1b). The results suggest nearly three-fold depletion of HNF4α protein in HCV-infected liver tumors.

#### Depletion of HNF4α leads to the induction of EMT markers

An important consequence of HNF4α depletion is the erosion of normal epithelial architecture and acquisition of mesenchymal markers, a hallmark of tumor progression [6, 19, 20]. We investigated the consequence of HCV infection-associated loss of HNF4α during development of liver tumors of humanized mice. HNF4α normally functions to maintain hepatic epithelial architecture by inhibiting the expression of epithelial to mesenchymal transition (EMT) genes. By contrast, loss of HNF4α promotes the expression of EMT genes and tumor progression. An earlier report described induction of EMT genes in HCV-infected human primary hepatocytes [21]. We describe results that build on these studies and show that induction of EMT in HCV-infected human hepatocytes is a direct consequence of targeting of HNF4α 3’-UTR by HCV-derived vmr11 RNA.

**Fig. 2:**
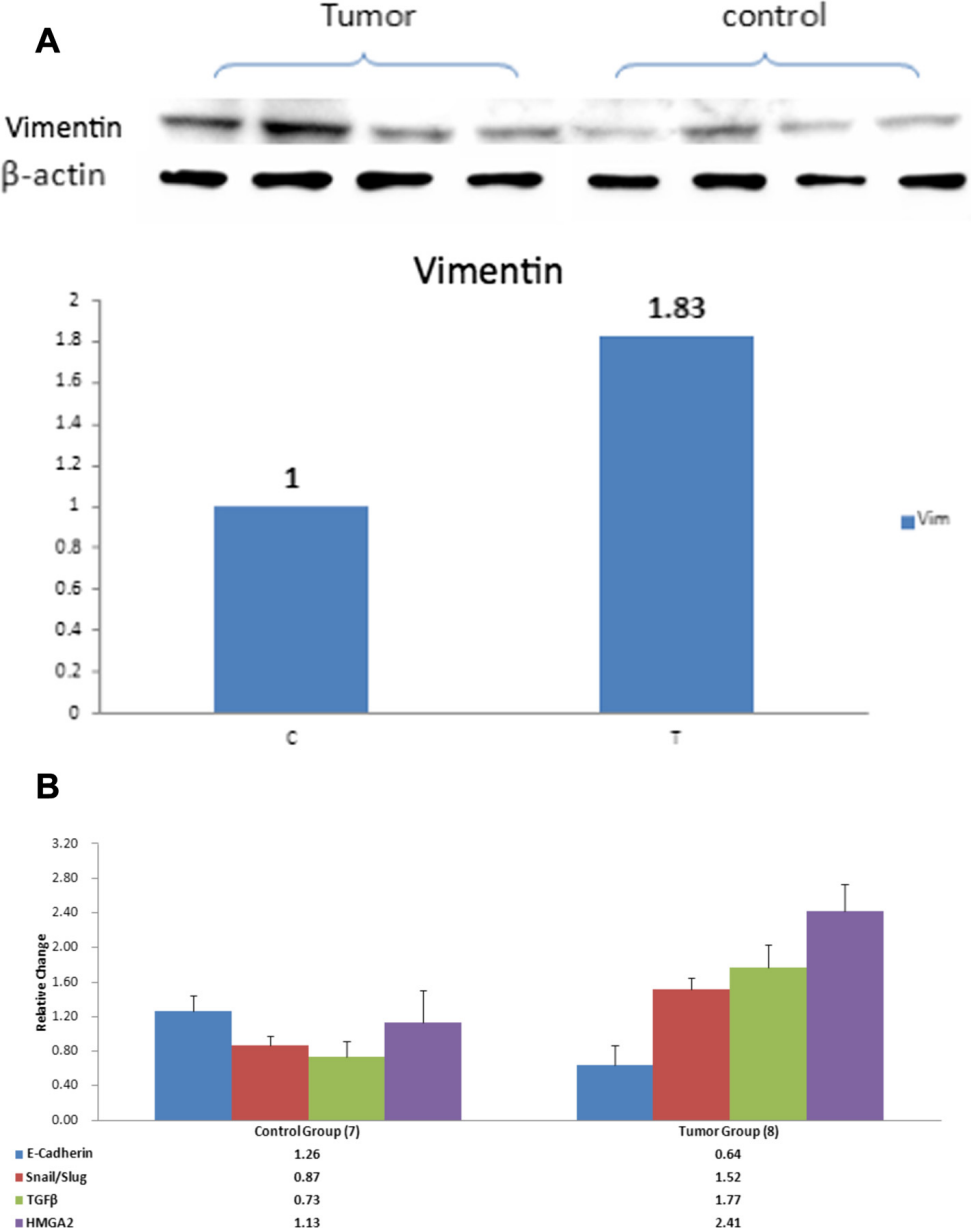
*Altered expression of Vimentin and EMT markers in liver tumors of HCV-infected MUP-uPA/SCID/Bg mice: (* ***2a*** *) Induction of Vimentin in liver tumor was compared to the liver tissue from human hepatocyte engrafted but uninfected chimeric mice. Representative Western blots of four tumors and four control liver is shown in the upper panel with* β*-Actin loading control. Lower panel shows relative increase in Vimentin in liver tumors was compared to the uninfected controls. (* ***2b)*** *Expression levels of EMT regulatory genes in liver tumors.* Liver protein from seven uninfected controls and eight HCV infected humanized mice were compared by Western blotting (run in triplicate*);* Relative values (Mean +/- SE, normalized to β-Actin loading control) of each protein is given underneath

Genes associated with epithelial-mesenchymal transition are critical determinants of tumor progression. Among the EMT master regulators, *Snail* is a strong repressor of E-cadherin, leading to the loss of epithelial structure and enhanced invasive properties of the tumor [12]. Vimentin by contrast is predominantly expressed in mesenchymal cells. We observed a marked induction of Vimentin in HCV-infected liver tumors compared to control liver tissues of human hepatocyte-engrafted but uninfected MUP-uPA/SCID/Bg mice (Fig. 2a).

HNF4α is known to suppress the transcription of EMT master regulatory genes, *Snail, Slug and HMGA2* [11]. *Snail* family of zinc finger transcription repressors regulate epithelial to mesenchymal transition (EMT) [22], partly through direct inhibition of E-cadherin [12, 23]. We investigated whether HCV infection-related loss of HNF4α promotes coordinated induction of EMT genes in human hepatocyte-engrafted HCV-infected SCID/Bg mice. Results (Fig. 2b) suggest that depletion of HNF4α in HCV-infected liver tumors correlates with the induction of Snail, HMGA2 and TGF-β and the suppression of E-cadherin expression.

#### Viral non-coding RNA, vmr11 targets HNF4α 3’-UTR

We explored the mechanism of HNF4α depletion in HCV-infected human hepatocytes. In an earlier report we described HCV-derived small non-coding RNA, vmr11 that displayed protooncogenic properties by blocking nuclear translocation of PTEN protein, and inducing γ H2AX, a marker of DNA double strand breaks [14]. Bioinformatics search of vmr11 targets based on the ‘PITA’ program [24] identified potential vmr11 target sequence within HNF4α 3’-UTR.

**Fig. 3:**
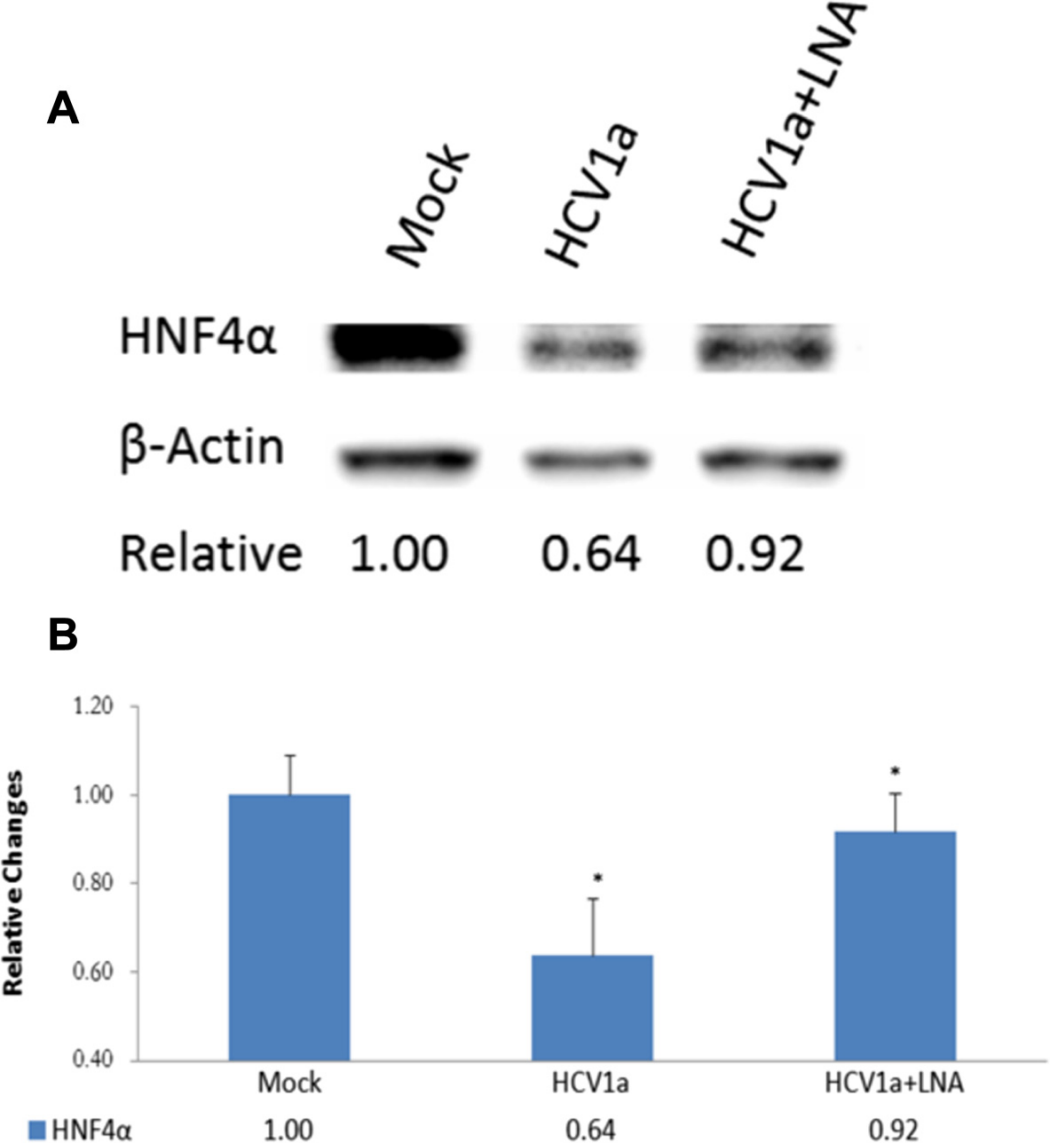
*Inhibition of HNF4*α *in human hepatocytes transfected with HCV genomic RNA*. Upper part (**a**) shows Western blots of HNF4α (from left to right), from mock transfected control cells, cells transfected with 1 μg HCV (H77) genomic RNA and (far right lane), cells transfected with 1 μg HCV RNA plus 50nM LNA-vmR-11antagomir. Cells were harvested 48 h post transfection. Lower part (**b**) shows relative change in HNF4α protein levels as compared to the mock-transfected cells (analyzed in triplicate Mean =/- SE, *p < 0.03)

**Fig. 4:**
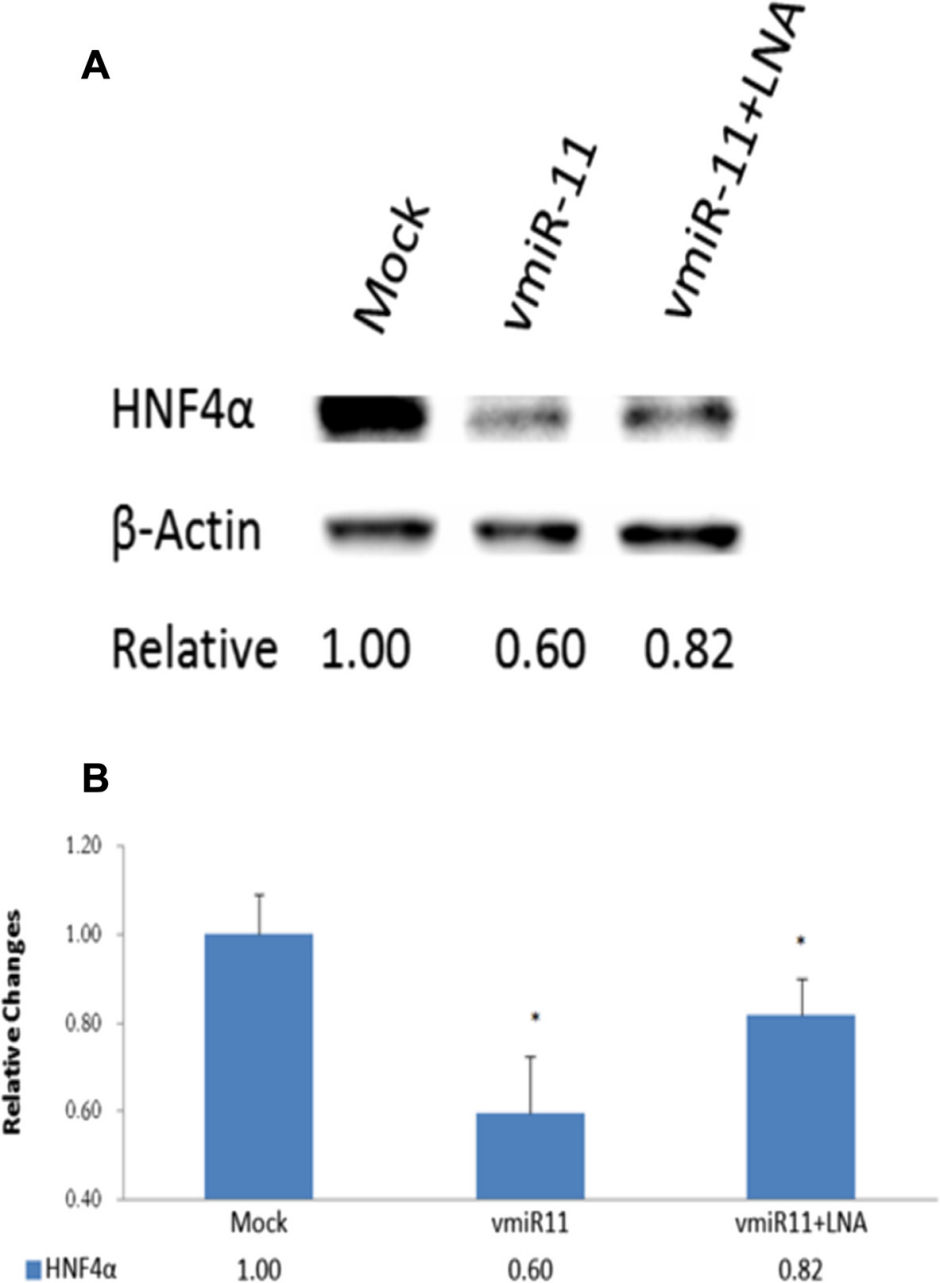
*Inhibition of HNF4*α *by vmr11:* Upper part (**a**), shows Western blot of HNF4α from control (mock transfected) cells, cells transfected with 50nM vmir-11 “mimic” oligonucleotides, and (far right lane), cells transfected with 50nM vmir-11 mimic plus 50nM vmir-11 antagomir. Transfections with vmr11 oligonucleotides were repeated at 24-h intervals. Cells were harvested at 48 h. Lower part (**b**), shows relative changes in HNF4α protein levels normalized to β-Actin loading control (*p < 0.03)

To assess a possible direct role of HCV-derived vmr11 in the depletion of HNF4α, we first determined the effects of vmr11 on HNF4α depletion in human hepatocytes transfected with HCV genomic RNA. Results (shown in Fig. 3) indicate that the loss of HNF4α protein in cells transfected with HCV genomic RNA is largely restored by the introduction of antisense vmr11 oligonucleotides. Interestingly, we observed similar decline in HNF4α protein levels of cells transfected with vmr11 oligonucleotides alone. Competition of the effects of vmr11 RNA *in vivo* by co-transfection of wild-type vmr11 and antisense vmr11 oligonucleotides restored HNF4α protein levels to that of mock-transfected control cells (Fig. 4). The results suggest that the loss of HNF4α protein, either in HCV replicating cells (transfected with HCV genomic RNA), or in cells transduced with vmr11 oligonucleotides alone, is due to the effects of vmr11 RNA. The results suggest that HCV-derived vmr11 RNA contributes to the loss of HNF4α protein in HCV-infected human hepatocytes.

**Fig. 5:**
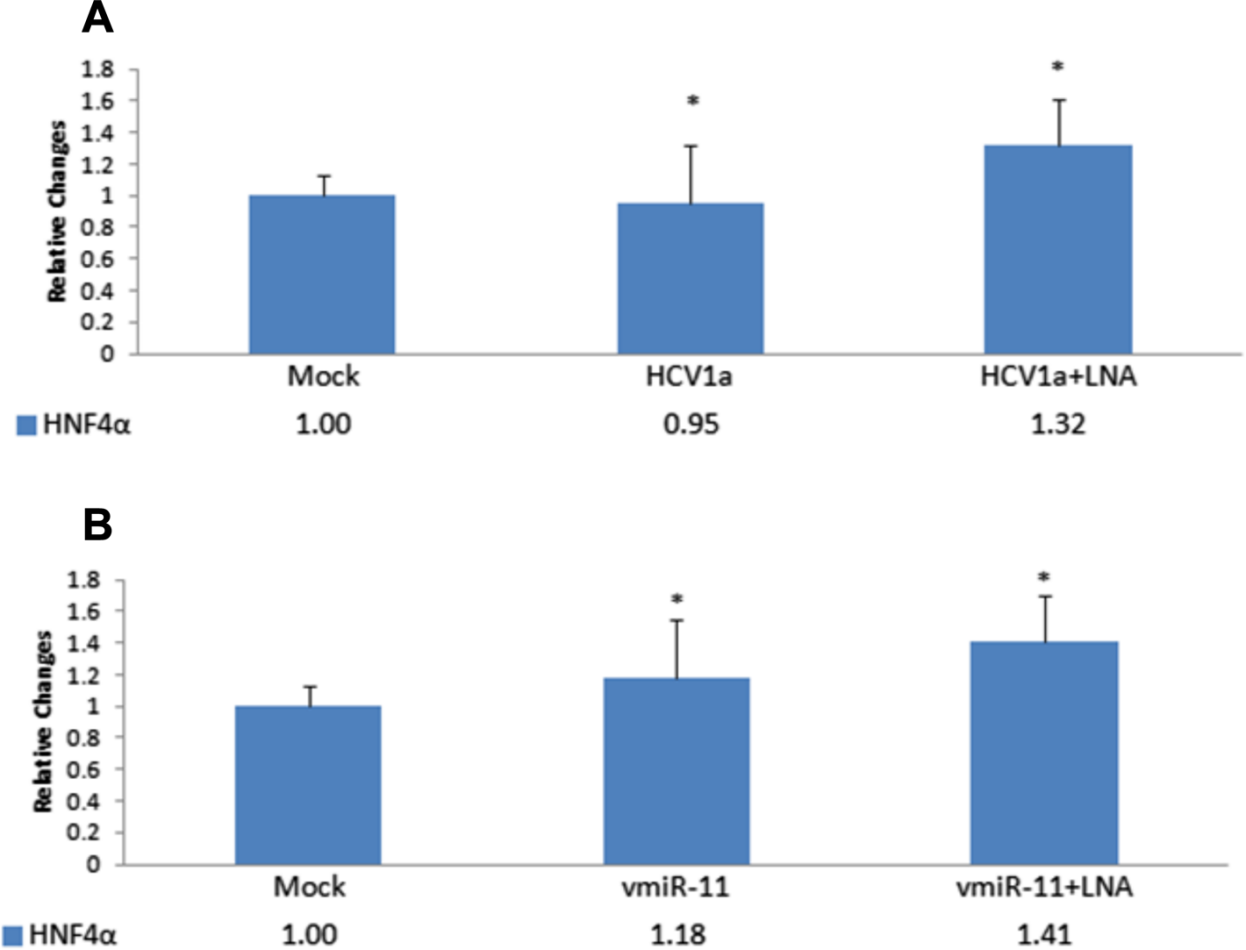
*HNF4*α *mRNA levels in HCV RNA or vmr11 transfected cells*. Upper panel (**a**): RT-PCR analysis of HNF4α mRNA from mock transfected, HCV (H77) genomic RNA transfected or HCV (H77) genomic RNA plus vmiR-11 antagomir co-transfected cells; Lower panel (**b**); RT-PCR analysis of HNF4α mRNA from mock transfected cells, or cells transfected with wild-type vmr11 (“mimic”) oligonucleotides or vmiR-11 mimic oligonucleotides plus LNA-vmr11 antagomir. The transfection conditions were as described in Fig. 4. Relative values (SEM) of HNF4α mRNA were estimated from three independent RT-PCR runs (*p < 0.01)

We then investigated whether the decline in HNF4α protein, either due to HCV replication or by artificially increasing intracellular vmr11 RNA, could result from the loss of HNF4α mRNA. We determined HNF4α mRNA levels in cells transfected with HCV genomic RNA or vmr11 oligonucleotides by RT-PCR. Results of HNF4α mRNA analysis (Fig. 5) indicate no significant loss of HNF4α mRNA, either in cells transfected with HCV genomic RNA or vmr11 oligonucleotides. This suggests that the loss of HNF4α in HCV-infected cells is initiated by post-transcriptional silencing of HNF4α by vial non-coding RNA, vmr11.

**Fig. 6:**
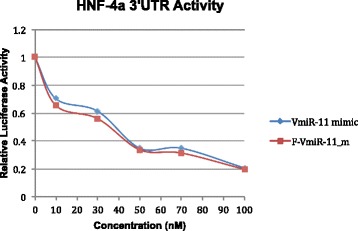
*Luc-HNF4a 3’-UTR assay:* Indicated concentrations of 22 nucleotide vmr11 “mimic” oligonucleotides or 2’-Fluoro modified vmr11 mimic oligonucleotides were co-transfected with Luciferase HNF4α 3’UTR reporter plasmid [13] and luciferase activity was quantitated three days post transfection as described

We further investigated whether the predicted vmr11 target site within HNF4α mRNA 3’-UTR is recognized by vmr11 RNA to block HNF4α expression. To do this, we determined the extent of inhibition of Luciferase-HNF4α-3’UTR reporter gene expression with increasing amounts of vmr11 oligonucleotides. To ascertain that vmr11-directed inhibition of HNF4α is not due to the instability of vmr11 oligonucleotides introduced into the cells, we used equimolar amounts of standard vmr11 oligonucleotides and 2’-Fluoro-stabilized vmr11 RNA. We co-transfected Luciferase HNF4α 3’-UTR reporter plasmid [13] with increasing amounts of either the wild-type vmr11 (“mimic”) oligunucleotides, or 2’-Fluoro-modified vmr11 oligonucleotides (TriLink BioTechnolgies). Luciferase reporter assays suggest that introduction of either normal vmr11 oligonucleotides, or 2’-Fluoro-stabilized vmr11 RNA was equally efficient in blocking HNF4α expression; about 75 % down regulation of HNF4α protein was observed, compared to cells transfected with irrelevant, scrambled oligonucleotides (Fig. 6).

#### Enhanced invasive properties of human hepatocytes transfected either with HCV genomic RNA or vmr11 oligonucleotides, can be counteracted by antisense vmr11

**Fig. 7:**
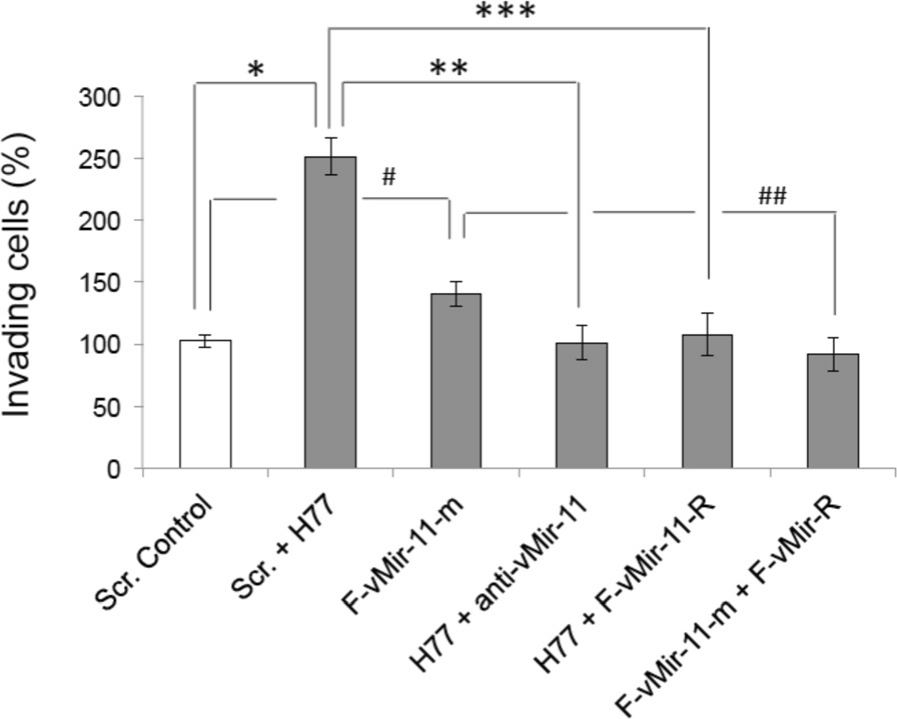
*Matrigel cell invasion assay*: (From left to right): Hepatocytes transfected with 50nM scrambled (Scr.) nonspecific control oligonucleotides, Scrambled plus H77 (HCV1a) genomic RNA, 2’-Fluoro modified vmr11 mimic (F-vMir-11-m), HCV 1a genomic RNA (H77) plus anti-vmr11 (anti-vMir-11), H77 plus 2’-Fluoro anti-vmr11 (F-vmr11-R), and Fluoro-vmr11-mimic plus Fluoro-vmr11-R. Three days post-transfection, the cells were processed for Matrigel assay. Data are mean + S.E.M. from 6 independent experiments for each bar graph. ^*^Significantly different from Scr. Control by ANOVA with post-hoc Dunnett’s test (*P* < 0.05)

Results suggesting that HCV-derived vmr11 RNA inhibited HNF4α expression, prompted us to ask if depletion of HNF4α is sufficient to promote oncogenic changes in HCV-infected human hepatocytes. We addressed the issue by *Matrigel* cell invasion assay by comparing acquired cell invasive properties of human hepatocytes transfected either with HCV genomic RNA (H77), or the 22 nt vmr11 “mimic” RNA (F-vmr11-m), (Fig. 7). Introduction of either HCV genomic RNA or vmr11 oligonucleotides resulted in significant enhancement of cell migration as compared to cells transfected with irrelevant, scrambled oligonucleotides. The cell-invasive properties induced either by HCV genomic RNA or vmr11 “mimic” oligonucleotides were reversed by introduction of anti-vmr11 oligonucleotides, suggesting that HCV-derived vmr11 RNA is responsible for promoting cell invasive properties of HCV-infected cells.

## Discussion

In a recent report [14] we described a negative strand HCV genomic RNA-derived small non-coding RNA (vmr11) and its ability to post-transcriptionally silence Transportin 2 (TRN2) expression. Transportin 2 interacts with PTEN tumor suppressor; TRN2-PTEN protein-protein interaction is required for nuclear translocation of PTEN. Significantly, nuclear insufficiency of PTEN in HCV-infected cells resulted in induction of γH2AX, a marker of DNA double strand break and genomic instability [14]. Such oncogenic properties of vmr11-regulated genes prompted us to investigate whether vmr11 target genes include other tumor suppressors that would be critical in promoting HCV infection-associated HCC. Bioinformatics search for vmr11 targets [24] suggested HNF4α tumor suppressor as a potential target of regulation by the viral non-coding RNA.

The results described here show post-transcriptional silencing of HNF4α by vmr11, and suggest an intriguing possibility that viral non-coding RNA initiated loss of HNF4α is sustained in HCV-infected humanized mice to promote HCC. The self-reinforcing circuit of hepatocellular transformation as reported in recent studies [13], indicated that siRNA-mediated transient suppression of HNF4α is sustained by establishing a miRNA feed-back loop without genetic mutation [25]. Here we explored the mechanism that triggers of HNF4α depletion *in vivo*. The experiments described here investigate whether the suppression of HNF4α and consequent induction of HCV infection-associated liver tumor development is regulated by viral non-coding RNA. We examined the loss of HNF4α *in vivo* in a humanized mouse model of HCV infection-associated HCC. We explored the mechanism of direct viral role in the onset of HNF4α depletion in HCV-infected human hepatocyte cultures.

Epithelial Cadherin (E-Cadherin) is the central target of *snail* and *slug* (also known as SNAI1 and SNAI2) transcriptional regulators. Epithelial cells express E-Cadherin, whereas mesenchymal cells express Vimentin [26]. During normal development and organogenesis epithelial to mesenchymal transition (EMT) is achieved by integrating a complex series of extracellular signals that are transient [19]. In tumor development the EMT-promoting stimuli are sustained by tumorigenic factors. The inhibition of E-Cadherin and induction of Vimentin are considered established markers of EMT and tumor progression. Our results suggest that progression of HCV infection-associated HCC is sustained by down regulation of HNF4α and consequent induction of EMT promoting snail/slug, negative transcriptional regulators of E-cadherin. EMT is also triggered by soluble growth factor such as TGF-β induced in parallel with snail/slug transcription regulators in liver tumors of HCV-infected chimeric mice.

The high-mobility group protein 2 (HMGA2) is a non-histone chromatin protein that is primarily expressed in tumors of mesenchymal origin. HMGA2 plays particularly important role in EMT maintenance of metastatic human lung and pancreatic cancer. Our results showing increased levels of HMGA2 in HCV infection-associated HCC of humanized mice are consistent with role of HMGA2 in the maintenance of EMT and liver tumor progression. Transforming growth factor-β (TGF-β is known to regulate the expression of EMT genes in cooperation with HMGA2 [27]. Our results showing coordinate induction of HMGA2, TGF-β along with the EMT markers are consistent with the model that viral non-coding RNA targeted depletion of HNF4α sets the stage for the development of HCC, tumor invasion and metastasis. Acquisition of a mesenchymal phenotype may allow HCV-infected cells to invade surrounding stroma and disseminate proliferative foci through the liver. These studies suggest the feasibility of testing antisense RNA-directed therapy of HCV infection-associated HCC.

Results described here suggest that epigenetic regulation of hepatocellular transformation is triggered by the inhibition of HNF4alpha, a direct taget of HCV-derived small non-coding RNA [14]. Our *in vitro* experiments with human hepatocyte cultures suggest targeting of HNF4α 3’-UTR by vmr11 RNA as the initiating event of HNF4α depletion in HCV-infected cells. Depletion of HNF4α in HCV infection-associated hepatocellular carcinoma promotes EMT genes and tumor progression. Targeting of HNF4α by viral small non-coding RNA more closely links HCV infection to hepatocellular transformation, and raises the possibility of antisense RNA-based therapy of HCV infection-associated liver cancer.
